# The Alphabet of Nanostructured Polypyrrole

**DOI:** 10.3390/ma16227069

**Published:** 2023-11-07

**Authors:** Sylwia Golba, Jan Loskot

**Affiliations:** 1 Institute Materials Engineering, University of Silesia, 75 Pulku Piechoty Street 1A, 41-500 Chorzow, Poland; 2 Department of Physics, Faculty of Science, University of Hradec Králové, Rokitanského 62, 500 03 Hradec Králové, Czech Republic; jan.loskot@uhk.cz

**Keywords:** polypyrrole, nano-organization, morphology, electrosynthesis, electropolymerization, drug delivery, sorption

## Abstract

This review is devoted to polypyrrole and its morphology, which governs the electroactivity of the material. The macroscopic properties of the material are strictly relevant to microscopic ordering observed at the local level. During the synthesis, various (nano)morphologies can be produced. The formation of the ordered structure is dictated by the ability of the local forces and effects to induce restraints that help shape the structure. This review covers the aspects of morphology and roughness and their impact on the final properties of the modified electrode activity in selected applications.

## 1. Introduction

There is a famous conducting polymers (CPs) triad that includes polythiophene, polyaniline, and polypyrrole (PPy). Among them, it is PPy that is highly attractive due to its wide range of applications. Its utilization spans outer-coating layers [1,2], sensors [3], drug-delivery sponges [4], charge storage in batteries [5], photothermal therapy in cancer [6], and electrodialysis [7]. The form of usage depends on the properties of the polymer and can be tailored to a large extent. It can be deposited as a protective thin layer for oxidizable metals [1] or as a powder [8] in chemical synthesis.

Electroactive conductive polymers can be oxidized (or reduced) by changing the electronic structure of the polymer backbone. The process is accompanied by a charge compensation event as a counterion moves into or out of a layer, forming a kind of ion-enriched sponge [9], an ion gate in the form of a membrane [10], or a hydrogel [11]. Polypyrrole is positively charged in an oxidized state and is neutral and hydrophobic in a reduced state. The ion movement possibility was utilized for the construction of potential controlled drug-delivery systems [4,12]. Many synthetic procedures with multiple ions were studied in this field, with salicylates [13], dexamethasone [14], or chlorpromazine [15] as examples. Drug release kinetics and efficiency served to relate the interconnections between synthetic procedure parameters and system work efficiency. The key parameters affecting the release kinetics of mostly ionic species were studied with the use of various analytical tools like fluorescence spectrometry [16], quartz crystal microbalance (QCMB) [15], or high-performance liquid chromatography (HPLC) [17]. Besides its electroactivity, PPy exhibits also antibacterial properties [8,18]. The tunable photophysical properties of PPy like photothermal conversion ability or Fenton catalysis ability allow for another emerging application, which is cancer therapy for tumor ablation and immune activation [19,20]. Photothermal therapy (PTT) utilizes heat generated locally by light-absorbing agents under near-infrared (NIR) laser radiation [20,21]. The photothermal potential of PPy particles for cancer treatment using NIR absorption was first demonstrated by Yang for material synthesized by aqueous-phase polymerization [22], where tumor growth was inhibited for the NIR laser irradiation (0.5 W/cm^2^) of the PPy treated samples. The bioinert surface of polypyrrole makes it a prospective contrast agent for photoacoustic imaging [23] studied with the different steric stabilizers of the dispersion polymerization like dextran (Dex) [24]. Smart scaffolds aimed at improving the functionality of the cardiac tissue were proposed by blending PPy into silk fibroin (SF) [25].

The coating ability of PPy makes it a suitable material for the modification of various substrates, imparting multiple functionalizations with prevailing “anti”- or “super”-type characteristics, like antioxidant [26,27], antibacterial [28,29,30], antifungal [31], superhydrophobic [32], anticorrosive [33], antistatic [34], anti-biofilm [35], anticancer [36], antitumor [37] properties. The application of intrinsically conducting polymers as new coatings presents the possibility of the re-passivation of pinholes in organic coatings [38] because of their inherent redox activity. They are also the base for the formation of smart self-healing coatings [2,39]. Protective polymeric film application for industrial substrates was thoroughly discussed by Saviour A. Umoren [40], mainly in terms of anticorrosion coatings and corrosion inhibitors, pointing to the challenges faced by the extended use of polymers for metal protection.

**Figure 1:**
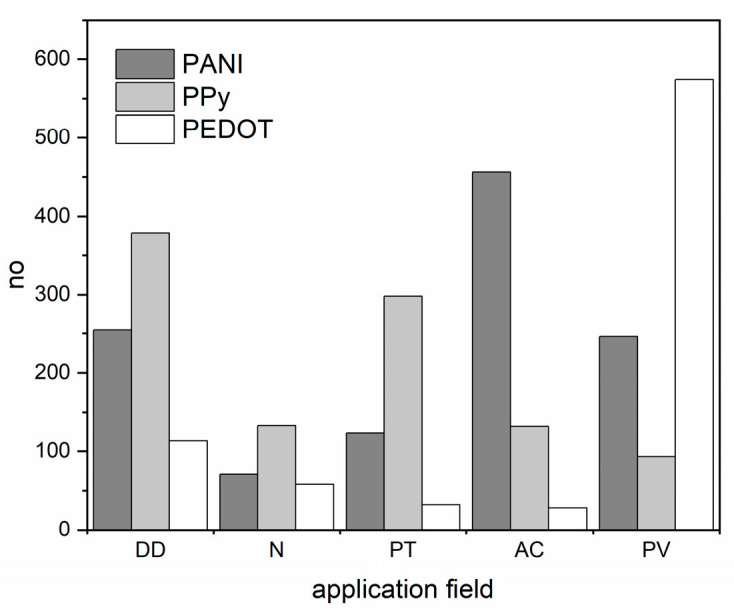
The comparison of the numbers of publications for PANI, PPy, and PEDOT concerning chosen fields of application namely DD—drug-delivery platforms, N—neural applications, PT—photothermal therapy, AC—anticorrosion protective coating, and PV—photovoltaic applications. (Source: Web of Science database, available on October 2023).

In the area of the application of various CP materials, namely poly(3,4-ethylenedioxythiophene) (PEDOT), polyaniline (PANI), and polypyrrole (PPy) there is constant competition to provide material with optimized performance. The issue is manifested in Figure 1, which presents a comparison of the number of publications concerning five chosen application fields, namely drug-delivery platforms (DD), neural applications (N), photothermal therapy (PT), anticorrosion protective coating (AC), and photovoltaic applications (PV) for all three polymers. Depending on the precise field, e.g., biomedical antibacterial material or charge storage material for battery construction, different aspects of CP identity are utilized [41]. Additionally, as we present in the current work, not only the chemical composition of the polymer chain but also the synthetic mode and procedure markedly influence the final material’s performance.

In this work, the impact of the morphology of PPy with topographical parameters like roughness on the physico-chemical properties of a material is reviewed in terms of its impact on perspective applications. The scope of the current work is dedicated mainly but not exclusively to electrochemically synthesized materials deposited in various electro-oxidation conditions, in the presence of templates as well as in template-free procedures, on various substrates. Applications are divided into biological and technological, with a further division into neural applications; antibacterial and implantable applications; drug-delivery platforms; sensors; and sorbents in the one group and corrosion protection systems; mechanical aspects; bubbles and nanoporous structures; carbon-based materials; and energy conversion systems in the second one.

## 2. Deposition of Electroactive Polypyrrole

Polypyrrole can be synthesized with various approaches using two main methods, namely chemical oxidative polymerization and electrochemical polymerization. For both methods, the template-based approach can be used to the induce nanostructural organization of the polymer [42], while one has to be careful not to destroy the previously formed organization at the template removal stage [43,44]. Also, other less common methods have been proposed, like radiolytic [45], sono-enhanced [46], or cell-assisted enzymatic processes [47].

Material prepared by the oxidation of the monomer with chemical oxidants (usually FeCl_3_ (either aqueous or anhydrous) [48], K_3_Fe(CN)_6_ [49], H_2_O_2_ [50], or an enzyme-mediated system [51]) is black powder. Both the yield and conductivity of the final PPy powder depend on parameters like solvent polarity, type of oxidant, pyrrole/oxidant molar ratio, duration, and temperature of the reaction [52]. Covering other materials with PPy coatings from chemically derived powder is problematic. The idea to overcome this obstacle was realized by polymer deposition from the gas phase [53] or by the preparation of composites with poly(N-vinylcarbazole) [54], poly(ethylene oxide) [55], polyvinyl chloride [56], poly(vinyl alcohol) (PVA), poly(vinyl acetate) (PVAc) [57], polyurethane [58], carbon black [59] or proteins like silk [60]. Other forms of materials containing PPy are also available, like substituted polymers, self-doped polymers, polymer/macroion materials, and hybrid materials (where the macroion is inorganic, polymeric, or of an organic blend) [61].

**Figure 2:**
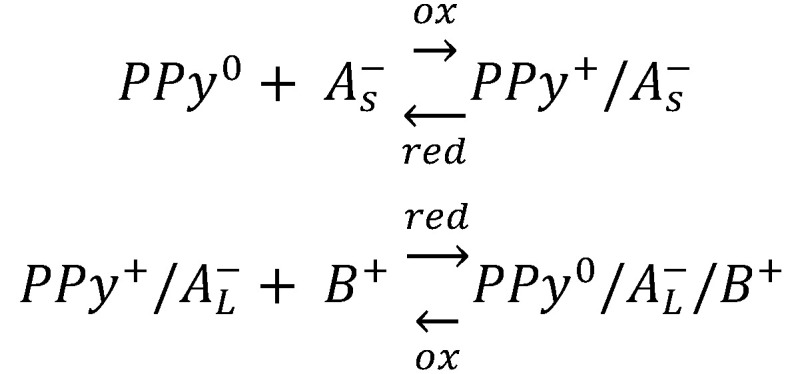
Redox reaction of PPy polymer in the presence of doping ions: A_s_^−^—a small mobile anion, A_L_^−^—a large immobile anion (reprinted from [65] with permission from Elsevier).

The electrosynthesis of PPy is initiated electrochemically, with the anodic oxidation of monomer leading to subsequent polymer formation. Concurrently, oxidation (doping) of the previously formed polymer occurs, as evidenced by the amount of consumed charge (2.07 to 2.60 F per mole of monomer with 2 F mol devoted to monomer oxidation) [52]. The electropolymerization mechanism has been thoroughly investigated [52,62,63] and involves several stages. The general process starts with monomer oxidation, followed by the coupling reaction, accompanied by the incorporation of the counterion. A charged polymer attracts anions to balance the charge. In the polymer formation process, both anions and electrons move through the film [63]. In the subsequent reduction, electroneutrality is restored by expulsion of the anions or by the incorporation of cations from the electrolyte solution. Upon the application of a positive potential, the neutral film is oxidized, and the anions are inhaled or cations are ejected (Figure 2). The redox activity of a polymer is governed by the electron transfer reaction and mass transport process [63]. The activity brings about serious structural changes manifested by conformation changes, swelling, shrinking, compaction, or relaxation [61]. For standard CP, de-doping is accompanied by the expulsion of anions along with polymer contraction [64]. In the case of anion immobility, movable cations penetrate the polymer to neutralize charge with observed expansion. In the work of Wallace, electrochemical atomic force microscopy (EC-AFM) was used to trace the dynamic actuation of polypyrrole films doped with polystyrene sulfonate [64]. The observation of actuation height displacement gave insight into factors limiting charge balancing processes, either of diffusion or current nature.

Electrosynthesis is a multi-step process where the yield and quality of the product are determined by factors like pH, electrode nature (e.g., material and shape), temperature, kind and concentration of monomer/counterion, applied procedure and potential value, solvent nucleophilicity, stirring conditions, and the presence/absence of gas bubble [62]. It is a sophisticated art to optimize and control all parameters in a single experiment. As the oxidation potential of pyrrole is lower (0.7–0.8 V) than that of other heterocyclic monomers and water, it is convenient to use it in the process [63,66,67]. At the beginning of polymer deposition, the reaction proceeds in the solution where oligomers are formed. As the chain length increases, they lose solubility, and the nucleation of PPy on the electrode surface occurs [52]. Most pyrrole units are linked at desired α-α (or 2,5) positions of the rings, yet irregular α-β or β-β linking is possible due to provoking cross-linking. This less advantageous coupling leads to the synthesis of soluble low-molecular-weight oligomers that worsen materials’ properties (e.g., reduce conjugation length and lower conductivity) [62].

The electrochemical method is advantageous for many reasons, including the straightforward formation of the electroactive film attached to the electrode surface in situ during the polymerization. Moreover, the process has a high yield concerning the consumed charge, which allows for the control of deposited mass and film thickness [43]. The working electrode for electrosynthesis is dictated by the intended usage and is frequently produced with materials stable at an anodic potential like Pt [68], Au [15], ITO-coated glasses (In_2_O_3_/SnO_2_) [69], FTO (fluorine-doped tin oxide) [42]. Also, the syntheses of PPy on stainless steel [70], Fe [71], Cu [72], Ni [73], Ti [74], NiTi [75], Ta [76], glassy carbon [77], graphite [78] or tungsten [79] were proposed. However, electrosynthesis on active metals competes with the metal dissolution possible in the relatively high oxidation potential condition [31]. The method used to overcome this problem is to cover the surface with a protective passive film before electropolymerization to prevent the dissolution of a substrate.

**Figure 3:**
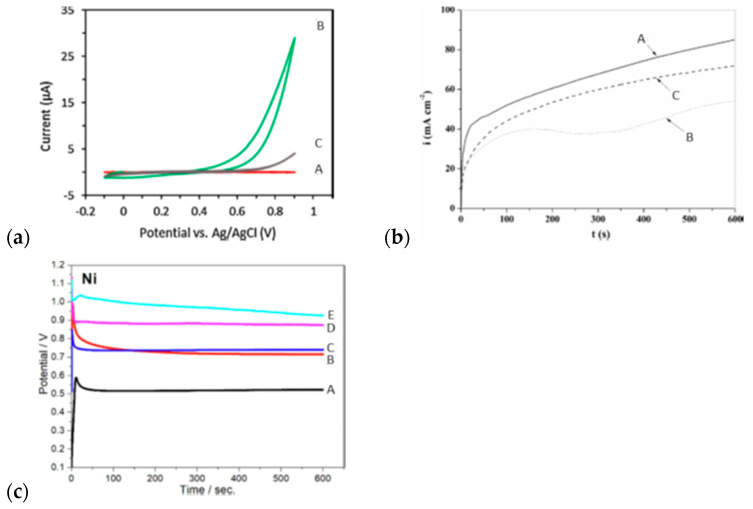
(**a**) Cyclic voltammograms of (A) ibuprofen (IBP) solution, the solution contains 0.005 mol/L IBP and 0.1 mol/L pyrrole with (B) 5 cycles, (C) 35 cycles (reprinted from [85] with permission from Elsevier); (**b**) chronoamperometric curves obtained for Ti-6Al-4 V alloy in 0.50 M Py + 0.50 M NaSa+x M ZnSO_4_, (A) x = 0, (B) x = 0.10, (C) x = 0.25 (reprinted from [86] with permission from Elsevier); (**c**) potential-time (E-t) curves for galvanostatic electropolymerization of pyrrole on Ni working electrode in (0.1 M C_7_H_5_NaO_3_ + 0.5 M Pyrrole) aqueous solution. Applied current densities: (A) 0.1, (B) 0.5, (C) 1, (D) 5 and (E) 10 mA/cm^2^ (reprinted from [87] with permission from Elsevier).

The external signal imposed to invoke oxidation may have different forms; hence, various electropolymerization techniques may be applied, including potentiostatic [80], galvanostatic [81], potentiodynamic [82], or pulsed [83] techniques (Figure 3). It was shown by Spinks that to obtain a material of high conductivity with superior mechanical properties, careful solvent-washing steps are required [84].

The properties of materials derived chemically and electrochemically are different. As shown by J. Joo [88] for chemically synthesized materials (dodecylbenzene sulfonic acid (DBSA) or naphthalene sulfonic acid (NSA)-doped), the density of states was markedly lower in comparison to electrochemically synthesized ones (PF_6_-doped). One dopant molecule was captured per three pyrrole rings in the PPy-DBSA and PPy-PF_6_ samples. At the same time, it was an electrosynthesis product that was more branched in the form of side chains or crosslinks (33%) than a chemical one (20%) [88,89]. The authors deduced that for the chemical technique, the use of large-size dopants was a crucial factor for the reduction in side chains or cross-linking. However, such a structure results in two opposite effects increasing solubility, also weakening the interchain interaction that reduces charge transport ability. A similar effect was observed by H. B. Li [90], where for chemically restricted synthesis in the presence of anionic spherical polyelectrolyte brushes (ASPB—modified SiO_2_ cores and poly(sodium-p-styrene sulfonate) (PSS) brushes), a more ordered (less-branched) polymer was formed. The electrical conductivity of PPy/ASPB nanocomposite exceeded approximately five times the value for pristine PPy powder (20 S/cm vs. 3.6 S/cm, respectively). Interestingly, a metal/polypyrrole (e.g., Pt, stainless steel) system was proposed as a quasi-reference electrode (QRE) for voltammetry in nonaqueous and aqueous environments [91]. High stability and reproducible potential gave rise to the perspective usage of this QRE, especially in the field of small electrodes utilized in nanocells.

## 3. Polypyrrole Doping and Conduction Path

CPs exhibit the optical and electrical properties of metals with the chemical properties of conventional polymers [92]. The conductivity of these materials comes from loosely bounded electrons in the backbone (as in metals) or from the doping process (as in semiconductors) [93]. For PPy, the conduction mechanism is strongly related to the motion of charge carriers named polarons and bipolarons (biradical cations) along the conjugation framework [62,93]. The oxidation level, manifested by the doping degree of the polymer, usually approaches 0.25–0.32 per pyrrole ring [52], depending on the kind and the charge of the inhaled anion. This means that one anion affiliates to 3–4 pyrrole units, accounting for 30–40% of the weight of the polymer [88]. There are an enormous number of ions utilized for doping PPy, including simple inorganic, monovalent ones like chloride [94]; organic ones like dodecylbenzene sulfonic acid (DBSA) [95] and *p*-toluene sulfonic acid (pTSA) [31]; large organic ones like sodium dodecyl sulfate SDS [82]; polymeric ones like polystyrene sulfonate (PSS^-^) [64]; DNA [96]; or polysaccharides like heparin [97]. The type and size of the ion induce a profound effect on the electronic, optical, and biomechanical properties of CPs [98], e.g., polymers prepared with pTS^-^ were shown to be more stable than PPy doped with ClO_4_^−^, BF_4_^−^ or NO_3_^−^ [56]. The changes in the electrolyte nature, e.g., passing from a small anion such as ClO_4_^−^ to a large polyanion such as poly(sodium-4-styrene sulfonate) NaPSS leads to the formation of thicker PPy–PSS hollow nanotubules [99].

**Figure 4:**
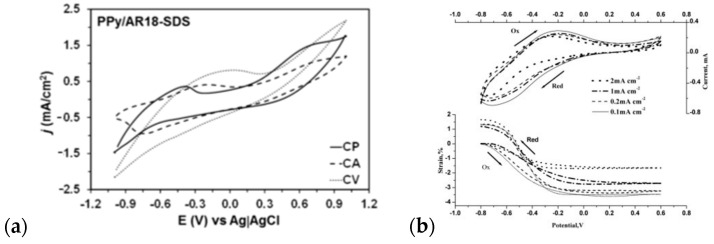
Cyclic voltammograms for (**a**) PPy/AR18 (azo dye) -SDS (prepared using chronopotentiometry (CP), chronoamperometry (CA), and cyclic voltammetry (CV)) (reprinted from [80] under the terms and conditions of the Creative Commons Attribution (CC BY) license (http://creativecommons.org/licenses/by/4.0/) (https://www.mdpi.com/2073-4360/11/11/1757 (accessed on 3 November 2023), (**b**) CV and ECMD curves during the second cycle for PPy/pTS polymerized at different current densities, and cycled in aqueous 0.1 M NapTS (reprinted from [81] with permission from John Wiley and Sons).

The relationship between polymerization rate and monomer concentration changes from linear for small doping anions to exponential for large doping anions such as PSS [100]. Large polyelectrolyte ions are firstly adsorbed on the electrode surface, thus retarding the monomer oxidation process. However, once the monomer oxidation is initiated, this process becomes much faster for increased polyelectrolyte concentration. The type of utilized dopant also influences the distribution of charge carriers in polypyrrole thin films, as shown by Pen-Cheng Wang [94] for chloride-, *p*-toluenesulfonate-, and anthraquinone-2-sulfonate-doped materials. The variation of dopant anion manifested in a change in the conductivity of thin films by three orders of magnitude (0.64 S/cm, 7.1 S/cm, and 120 S/cm, respectively) [101]. The exemplary CV curves recorded during the doping of the PPy material are present in Figure 4.

The anion incorporated into the polymer at the stage of synthesis also influences the thermal stability [62]. It was pointed out in many works that the thermal stability of PPy should be improved, especially in oxidative atmospheres such as air [62]. Additionally, research impact on the enhancement of mechanical properties is urged for prospective applications. The aspect of aging polypyrroles derived by the chemical method was discussed by Mičušík [102], who reported that during the tests, -N-C=O carbonyl groups were formed after oxygen attack, mainly at the α position of the pyrrole unit. SO_4_^2−^- and S_2_O_8_^2−^-doped materials had shorter conjugation lengths, owing to the interaction of sulfate groups with the polymer chains to create sulfonic functional groups. The use of anionic surfactant (DBSA) as a co-dopant improved the stability in ambient air [102]. Also, Kopecký [96] studied the reversibility of protonation/deprotonation cycles for nanotubular polypyrrole taking into account the long-term stability. They reported that deprotonated samples aged faster, while reprotonation by acids improved the stability [103].

Thinking of the technological application of PPy also overoxidation process should be taken into consideration [104,105]. Profound changes in material properties may occur after being exposed to oxidizing conditions or very positive electrode potentials [104]. It was proved that the hydroxyl radicals formed during water oxidation are responsible for PPy oxidative degradation [105]. The changes lead to the irreversible depletion of the electroactivity, also resulting in the decreased diffusion of ionic species. Still, it may be beneficial for some applications such as protection against electrode fouling, provision of permselectivity, action as a host or cover for immobilized reactants, and provision of the material for molecular imprinting [96,104,106]. Several methodologies were proposed to inhibit overoxidation, including the control of electrode potential, forming copolymers, supplementation with radical scavengers, and a new synthesis medium (e.g., ionic liquids) [104,105].

**Figure 5:**
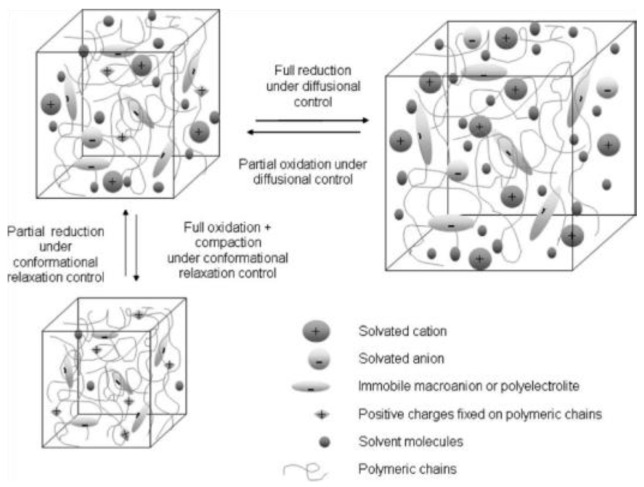
Scheme of the reversible changes of volume associated with the doping-dedoping cycle in CP blend (reprinted from [107] with permission from Elsevier).

The reversible variation of volume associated with the electrochemical reduction–oxidation processes was studied by Otero [107]. If a polyelectrolyte or an organic macroanion is incorporated into a CP, then the electrochemical process induces changes in free volume, which is a counterbalance of two effects, namely electrostatic repulsions between immobile macroanions and the exchange of ions and solvent molecules between the polymer and the solution (Figure 5). A polypyrrole/p-toluenesulfonate blend on Pt substrate films shrunk under anodic potential, while cathodic reduction and swelling were observed to be governed by relaxation–nucleation kinetics [107]. Such changes are adopted for artificial mussel construction in a trilayer or bimorph configuration [108].

The oxidation of polypyrrole depends on crossing the activation energy, which includes two components—chemical activation energy and the energy connected to the relaxation of the polymeric structure induced by the entrance of counterions. The value of the second component relates to changes in the film’s molecular structure during the process and film thickness [109]. Conductivity relaxation and charge transport mechanisms in polypyrrole nanofibers were investigated by dielectric relaxation spectroscopy [110], leading to the conclusion that the mechanism is dominated by the hopping of trapped charges. Film thickness is also the factor for the control of release rate in controlled delivery systems [111] when applied as a matrix layer or a protective, diffusion-controlling cover.

## 4. Morphology of Polypyrroles

**Figure 6:**
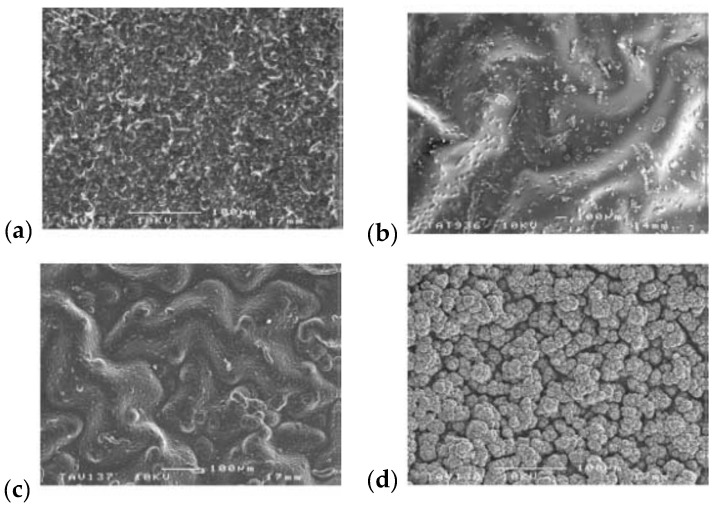
Surface topography of polypyrrole (SEM images) generated in the presence of the respective counterions: (**a**) chloride, (**b**) polyvinyl sulphate, (**c**) dermatan and (**d**) collagen (reprinted from [112] with permission from Royal Society).

The morphology of material pictured by the SEM or optical images is the result of the events that occur at the lower stages of organization. The mutual inter- and intramolecular interactions silenced or strengthened by the chemical composition of the reaction environment, synthetic procedure, or post-synthetic modification lead to the formation of specific arrangements. The surface morphology alone cannot sufficiently characterize the whole polymer layer structure, but it is the outfit of the event that takes place within the macrochains and between participants of the synthetic process. The knowledge provided by studies of surface morphology and its origin can provide suitable methods for the synthesis of films with required properties in reverse engineering mode. For a typical polypyrrole, a usual cauliflower morphology is known. However, through the manipulation of the available factors, it can be significantly altered for materials deposited in the presence of different counterions. When adjusting synthesis duration, the shorter times produced thin films of similar characteristics. The extended time provided thicker films with distinct topography, which proved that counterions influence topography (Figure 6) [112]. 

It is interesting as one-dimensional (1D) nano-structure materials extend the application areas in comparison to bulk ones, which are frequently composed of spherical particles. The 1D morphology of the particles lowers the percolation threshold and increases the specific surface area. This leads to higher electrical conductivity along with improved stability. Such products are usually characterized by uniform morphology, low polydispersity, and a high aspect ratio. It was found that the nanostructured organization depends on the polymerization rate and can be supported by the use of additives like a steric stabilizer (polyvinyl pyrrolidone (PVP)) [113].

Several electrochemical methods for the synthesis of nanostructured PPy were reviewed by Bocchetta [114] along with template-based or template-free polymerization. The role of hard templates (like ZnO nanorods, α-Fe_2_O_3_ nanowires, silica and silicon-based templates, anodic TiO_2_ nanotubes, and colloidal crystals) were elegantly presented. Also, several methods for self-assembly template-free methods (like surfactant micelles, gas bubbles, or azo dyes complexes) were delivered [114]. The main parameters that govern the ability to self-assemble PPy nanofibers in electrodeposition (template-free process) are pH, applied potential, monomer type, and doping ion concentration. Diverse interfaces and morphologies of polymer nanodeposits were formed, and plausible mechanisms of their formation were discussed. The composition of solution and the formation of weak interactions between molecules are the leading forces that impose on the self-alignment ability of PPy [114]. A similar effect was found for cone-shaped PPy deposited on an Au substrate in a concentrated monomer solution (concertation up to 0.6 M) [115]. An elegant synthesis of porous PPy was proposed by Cysewska [116], with a platinum screen-printed electrode used as the substrate. With the increase in deposition charge, the fibers of various thicknesses and cup-like structures were formed, as shown by SEM images. The authors correlated the height of the 3D structures with the electroactive surface area (A_eff_) of the polymer and showed a mutual increase with a growing deposition charge. The observed 3D cup-like structures were formed based on the thick fibers grown at the pristine stage [116]. PPy nanowires (PPy-nw) were successfully deposited by del Valle [117] by the use of a mesoporous silica template. A brush-type conformation of nanowires was discovered with wires sized to 1970 nm in average length and 30 nm in diameter. Reproducible PPy nanowires provided enhanced charge-discharge characteristics supported by improved adhesion to the substrate.

Having acknowledged the core information about the types of morphologies, we proceed to discuss the application fields and provide an overview of the influence of morphological parameters on their performance.

### 4.1. The Impact of Morphology on the Bio-Applicability of PPy

#### 4.1.1. N as Neural Applications

Conducting polymers exhibit ionic and electronic conduction and resemble the mechanical and conductive properties of living organisms. This makes them interesting materials for application in the bioelectronic field [118]. They are proposed as coatings of neural and osteogenic implants for signal recording and electrical stimulation. The idea is based on the ability of a material to overtake high-efficiency signal transduction at the interface while staying ion-permeable. Some strategies were proposed for such implants to enhance cell−substrate and cell−cell interactions, providing an artificial matrix resembling ECM (extracellular matrix) behavior in terms of chemical, topographical, and mechanical properties. Histological analyses of tissue surrounding polypyrrole-based implants in rats showed an immune cell response similar to poly(lactic-co-glycolic acid), which is an FDA (Food and Drug Administration)-approved material [119]. For PPy-based sciatic-nerve guidance channels implanted in rats, low inflammatory responses were reported [120]. Substrate roughness and chemical functionalization are useful tools to adjust mimicry strategies on CPs [118]. However, the implants often fail due to bacterial infection [121]. Working on conducting materials in living organisms requires mindfulness as imposed electrical signals induce multiple effects on the cells, like the rearrangement of the cytoskeleton, the depolarization of the plasma membrane, the alternation of protein conformation, or the modulation of membrane ion influx [92]. Moreover, the drawback of CP applications lies in their inherent inability to degrade [92]. The way to overcome this handicap is to produce blends with biodegradable polymers like chitosan [122], gelatin [123], or polylactide (PLA) [124]. The work of Liang provided an overview of the materials that were integrated with PPy [125].

Effective neural interfaces require materials able to convert neural signals to digital ones. A list of desired material properties was delivered by Krukiewicz covering low electrical impedance accompanied by high cathodic charge storage capacity, high charge injection capacity, and electroactive surface area, with adequate mechanical characteristics [126]. The formation of responsive, durable, and selective implantable bioelectrodes is the aim of many research teams in the field of bioelectronics applications [125,127,128]. Available electrodes are formed with mechanically hard metallic materials that do not fit with biological tissue that is soft, ionically based, wet, and dynamic. Their interaction leads to reactive tissue responses and electrode encapsulation. The low surface area of these electrodes reflecting the planar microscale geometry translates into disadvantages of the application, low signal-to-noise ratio, high impedance, and low charge injection capacity at electrode–tissue interfaces [129]. There are multiple ideas for overcoming these obstacles by optimizing size and shape, choosing substrate material, bioactive coating deposition, or delivering drugs. The performance of devices can be also improved by the organization of PPy at the nano level [114]. Decreased distance to transport ions along the chains converts into enhanced electrical conductivity and the reduction in the impedance at the electrode/electrolyte interface. Through nano-organization, deposited materials induce electrical advantages by increasing the surface area related to nodular morphology, which elevates resistance to the mechanical stress derived from the electrochemical half-reaction. The neural tissue performance is dependent on physical properties imposed by the topography, roughness, or mechanical rigidity of materials [98]. Hence, many features of the conducting polymers authorize their usage in neural tissue engineering. A neural device is designed for long-term service and sensitive communication with the neural network. To work properly, the material interface should resemble the properties of the tissue. Morphology-dependent electrochemical stability for composite coatings was reported by Zhou [79]. The composites were based on polypyrrole/nano-ZnO electrodeposited with different protocols on tungsten substrates. Coated samples showed safer capacitive charge transform behaviors with increased cathodic charge storage capacity (CCSC) and safe charge injection capacity (Q_inj_) for samples deposited with cyclic voltammetry (CV), in comparison with pure substrate. In addition, produced coatings enhanced long-term electrochemical stability, preventing delamination or cracks on the surface [79]. Such behavior is advantageous for providing a stable interface between electrodes and neurons during deep brain stimulation treatment. The morphologies of the conductive fiber scaffolds (CFS) of PPy on glucose–gelatin fibers induced properties that led to a material characterized by linear actuation in combination with dual sensing capability. Such materials can be applicable in fields like soft robotics, smart textiles, or e-skin [130]. Samples actuated in organic and aqueous electrolytes consisted of fiber scaffolds (32 μm thickness) composed of randomly oriented fibers, with single fiber diameters of around 0.8 μm. The PPy coating covered individual fibers separately, resulting in a uniform layer of 0.3–0.4 μm thickness. Polypyrrole/poly-L-lactic acid (C-GO/PPy/PLLA) films enriched with carboxylic graphene oxide were fabricated with an electrochemical deposition step by Xianchun Chen [131]. The immersion test (4 weeks) showed the stability of the conductivity and tensile strength of this material. It was prescribed to the hydrogen bonding between graphene oxide’s carboxylic groups and pyrrole’s imino groups. A conduit supported with electrostimulation was used to successfully bridge a 10 mm sciatic nerve defect in rats [131]. The average diameters of composite fibers immersed in PBS decreased with time. It was faster for C-GO/PPy/PLLA, pointing out that PLLA fiber cores degraded more rapidly in the material with a bigger surface area and pore volume. PPy (dodecyl benzene sulfonic acid (DBSA) as dopant) on gold-coated mylar, deposited galvanostatically (current density of 0.1 mA/cm^2^ for 10 min), was used to counter the impaired neurite outgrowth of primary pre-frontal cortical (PFC) neurons from mice [132]. The use of polymer-mediated electrical stimulation prevented the reduction in neurite outgrowth and related synaptic protein expression in the primary PFC neurons, providing the usability of the technique in treating neurodevelopmental diseases [132]. A multi-block conductive nerve scaffold was proposed by Zheng [133], where PPy was deposited on the nanofibers of bacterial cellulose (BC). Electrical stimulation was assessed by glucose oxidation and oxygen reduction. The mean neurite length of dorsal root ganglions cultured on the composite composed of the Pt-BC/PPy-N-CNTs scaffold was significantly longer (55%) in comparison to BC/PPy cultured ones. The composite scaffold also promoted nerve regeneration [133]. Khorrami produced hollow polypyrrole microcontainers with electrosprayed poly(lactic-co-glycolic) acid (PLGA) microspheres, which were used as degradable templates [129]. The effective surface area of the gold electrode increased markedly along with the increase in deposition charge density. This was accompanied by a profound decrease in impedance value (91%) and an increase in charge storage capacity (85%) in comparison to uncoated gold electrodes.

#### 4.1.2. A as Antibacterial and Implantable Applications

The antibacterial behavior of PPy depends on a diversity of structural parameters such as surface area, aggregation level, and additive (e.g., metal nanoparticles) incorporation [8,29]. These properties are related to polymerization solution compositions and conditions; hence, the final material characteristic is a compromise between them [134]. For example, electrochemically deposited PPy with p-toluenesulfonate (TsO^−^) dopant is highly conductive [135] but still does not show enhanced antibacterial activity [31].

The real battle takes place locally; hence, the core antibacterial inhibition mechanism is relevant to the interaction between the atoms of the biomaterial and the bacteria. The mechanism relies on an electrostatic interaction between the positive charges located on the polymeric chains and the negative charges located on the membrane cell of the bacteria [3]. The attack on the cell wall of the bacteria by the charged N atom and dopant ions of the polymers is possible. Changes in the preparation procedure impact the parameters modifying its resilience, e.g., by controlling the sizes of PPy nanoparticles. The electrostatic interaction of polymer nanoparticles with bacteria leads to bacterial cell death. The characteristic time-kill of bacteria in contact with the chosen agent proved the superior bactericidal activity of highly soluble PPy with a minimal period of interaction to inhibit the growth of bacteria like *E. coli*, *K. pneumoniae*, and *S. aureus* [8]. The PPy effect on *P. zopfii* cells (saprophyte microorganisms involved in the occurrence of infections) was verified by Ely [136] with an evident decrease in the number of cells after treatments with sublethal doses of PPy, both in planktonic and sessile forms. After the evaluation of the effectiveness in vivo, a formulation could be prepared to treat the animals naturally affected by *Prototheca* spp. [136]. Smart antimicrobial material with dual functionality was prepared by Děkanovský [137] by dosing stretchable polydimethylsiloxane (PDMS) with polypyrrole. The composite was found to be superhydrophobic with self-cleaning ability. The presence of a conductive additive provoked the ability to electrically trigger the release of an immobilized model drug (namely crystal violet). Focusing on morphology, it is visible that the mixing of components markedly increases the surface roughness, forming typical aggregates. The antimicrobial protection tests in the *E. coli* solution showed a lack of bacteria adhesion on the surface, while applying electric field-induced interaction with the bacteria was accompanied by changes in the sample morphology [137]. A duplex coating formed potentiostatically on a magnesium alloy (AZ91D) was studied by López [138,139] in a simulated physiological environment. A dual system composed of a pristine protective layer deposited in molybdate solution and covered with the outer layer of PPy film was electrosynthesized in a solution of sodium salicylate. Morphologically, a single layer of PPy hollow rectangular microtubes was observed for an electrolyte solution of high concentration (0.50 M), while a typical globular arrangement was found for one formed at lower concentrations (0.10 M). The morphology of the bilayer coating presented the globular structure on the electrode surface. The bilayer was modified by the ingress of silver ions, which imparted antibacterial properties [138] more profound for the higher-surface-area microtubular polymer. Polypyrrole deposited on carbon steel using several sulfonic acids as dopants was tested to set the effect of acidic dopants on passivation [31]. The biocidal activity for electrodes with a polypyrrole coating increased markedly, similar to the coating doped with SDBS, which caused a 5.66 log reduction in bacteria within 10 min, considered as a 100% killing of bacteria. This biocidal activity was based on both the destruction role of the sulfonate group and the length of the chain attached to the sulfonate [31].

A multifunctional polypyrrole/zinc oxide (PPy/ZnO) composite was deposited with the CV method on Mg alloys [140] with perspective applications in the field of orthopedic implant materials. Based on the result of an in-vitro test, improved adhesion and proliferation of cells were confirmed. This was accompanied by significant antibacterial ability against *E. coli* at the level of 96.5 ± 2.6%, with coarse and wrinkled bacteria cells [140]. In the search for the better osseointegration of titanium-based implants, a new lanthanum-substituted hydroxyapatite(HAP)/poly(N-methyl pyrrole) (pNMPy) coating was proposed by Mathi [141]. Interestingly, N-methyl pyrrole was used to increase the hydrophobic effect by methylation. The bilayer surface morphology revealed granular and cauliflower-like microspherical structures with reduced size. The presence of an adlayer of HAP tuned the morphology into a microstructured flower [141]. The adherence of the coating to the titanium substrate was estimated as perfect, with small pores beneficial for subsequent cell attachment. The result of antibacterial activity studies against *S. aureus* and *E. coli* showed that a decrease in bacterial colonies was noticed for the La-HAP/pNMPy bilayer, proving its resistance to bacterial infection.

**Figure 7:**
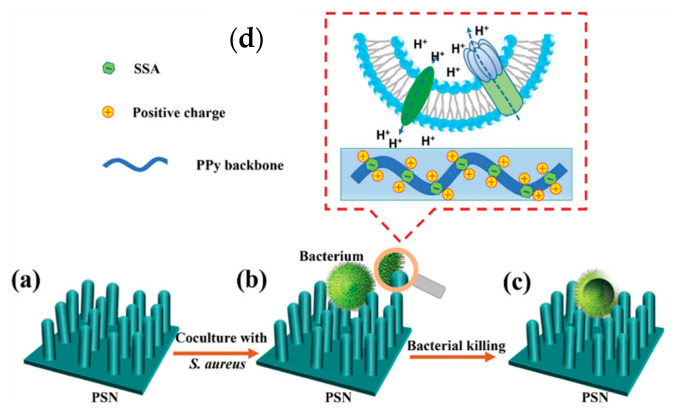
Mechanism proposed for interaction between SSA-doped PPy nanorods and the bacteria: (**a**) scheme of the substrate, (**b**) bacteria adhered to the nanorods after co-cultured with *S. aureus*, (**c**) possible cell death, (**d**) interaction of PPy backbone and bacterial wall. (reprinted from [142] with permission from Royal Society of Chemistry).

Nano-functionalized polypyrrole with high surface potential was the subject of the study of Zhou [142]. In the template-free procedure, sulfosalicylic acid (SSA) was used to assist in the ordering of PPy macrochains and to modulate the surface electrical properties of the coating. SSA-doped PPy nanorods were successfully built on a titanium substrate with a diameter of approximately 100 nm, increasing specific surface area markedly. The material was proved as an antibacterial in comparison to irregular PPy/Cl, which was prescribed to two factors—nanorod morphology and high surface potential induced by the dopant [142]. The possible mechanism of interaction between the material and the bacteria is shown (Figure 7). A negatively charged dopant (sulfosalicylic anion, SS^−^) increased the PPy surface potential, as shown by SKPM (scanning Kelvin probe microscopy). Also, increased specific surface area enhanced the measured surface potential of PPy.

Potentiostatically synthesized polypyrrole deposited by Martinez [86] on Ti-6Al-4V alloy is aimed at dental implant applications. Zn particles were immobilized either during or after the process within the microstructured matrix, with the second method being more effective in terms of antibacterial activity. SEM micrographs of the coatings presented hollow rectangular-sectioned microtubes of polymer deposited in the presence of salicylate ions. The organization event relates to the precipitation of rectangular structures of salicylic acid triggered by the decreased pH, with subsequent polymer deposition at the walls of the formed crystals [143]. The addition of ZnSO_4_ to the solution for PPy/Zn formation disturbed the tube’s formation, leaving them with fringes at the ends. Such action is induced by the precipitation of zinc salicylate Zn(Sal)_2_ during the electropolymerization process [86]. There were no cracks or products of corrosion detected on the films after testing by immersion in artificial saliva solution.

The coating of a carbon steel surface with PPy, aimed at improving corrosion resistance along with antimicrobial properties, was proposed by Jaouhari [144]. PPy films synthesized galvanostatically reproduced compact distribution with globular components of sizes in a range of 2 to 10 nm. The structural changes between the films were imposed by a deviation in the polymer growth mechanism. It was proposed that the implosion of the cavitation bubbles on the surface of the electrode produced many nucleation sites. The antibacterial activity quantification proved high activity for PPy-coated steel with silver, accompanied by reduced Fe^2+^ ion release [144]. Polypyrrole films embedded with copper cations were deposited on 316 L stainless steel and tested as a water disinfection system [145]. SEM images of a PPy-Cu-coated electrode revealed PPy microtubes that were not damaged after examination in the lab-scale continuous flow tests system, and they were not adsorbed with the bacteria cells.

The electrosynthesis of polypyrrole on nitinol proposed by Saugo [75] led to materials for which morphology was influenced by the electrolyte (sodium salicylate, NaSal) concentration. For a low concentration of NaSal (0.10 M), the standard globular morphology was obtained, while the increase in concertation (to 0.50 M) changed it into hollow microtubes of rectangular shape. These hollow tubes were utilized for silver immobilization within the PPy matrix, with quantitative dependence on the polymer oxidation degree [75]. The antibacterial activity of the coating was manifested in the test against the Gram-positive *Staphylococcus aureus* and *Staphylococcus epidermidis* bacteria. 

An interesting feature of PPy is also its antioxidant activity, which predisposes the material for application as a protective barrier. In the studies of Hsu [146], the antioxidant activity of PPy was established for chemically synthesized powders (ammonium persulfate (APS) as an oxidant). The ability to scavenge free radicals was tested in a reaction with the stable DPPH (1,1-diphenyl-2-picrylhydrazyl) free radical. The study of the impact of the diameter of PPy nanotubes on antioxidant activity was proposed by Kumar [27] for a material obtained in the reactive self-degrade template process. In the synthesis, it was a cationic surfactant, namely cetrimonium bromide (CTAB), that was used to ensure control over the diameter of the nanotubes. The concentration of model radicals declines with a decrease in nanotube diameter [27]. It showed enhanced antioxidant activity with a decrease in the diameter of nanotubes, which implies that there were more available reaction sites for DPPH free-radical scavenging. Highly soluble polypyrrole was reported as a bactericidal agent [8]. Its activity was positively verified when directly incorporated polymer nanoparticles induced bacterial death. The results of the characteristic time-kill of bacteria in contact with bactericidal agents proved superior performance for highly soluble and branched polymers [8].

The bioactivity of 316 L stainless steel covered with a polypyrrole/hydroxyapatite layered hybrid multilayer system was reported by Etminanfar [147]. The morphology studies revealed that in the presence of a PPy interlayer, the porosity decreased and changed the plate-like surface morphology of Hap. Rose-like aggregates were found throughout the microstructure of the bilayered deposit. Such polymer-ceramic layered hybrid systems were proposed for orthopedic applications [147]. The morphology influenced the antibacterial performance of the PPy-based composite where cellulose nanopaper (CNP) was enriched with chitosan (CS) [148]. The composite surface was smoother with CS located on the surface and at the interior lamellar structure, while the presence of PPy (CNP/CS/PPy) increased the roughness and density of the interlayer structure. The antibacterial performance was higher for the PPy- or CS-loaded system and superior for the CNP/CS/PPy one as a result of the synergy effect [148].

#### 4.1.3. D as Drug-Delivery Platforms

**Figure 8:**
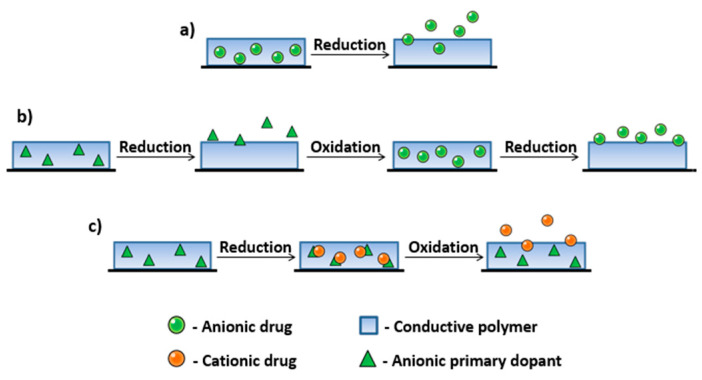
Drug loading and release mechanisms in CPs: (**a**) one-step loading (for anionic drug); (**b**) three-step loading (for anionic drug); and (**c**) loading of cationic drug (reprinted from [12] with permission from Elsevier).

Drug-delivery systems constructed on CPs use the polymers’ ability to electrical switch between an oxidized and a reduced state, accompanied by the uptake or expulsion of charged molecules from the bulk of the polymer [12]. Drug loading can be realized in several ways depending on the type of the molecule: a one-step immobilization procedure for small anionic compounds, such as dopants; a three-step method where the synthesis and drug loading are separated; and a modified three-step method for cationic drugs (Figure 8).

The release of such substances from ICP matrices is governed by electrostatic forces [149] accompanied by expansion and contraction movements induced by the electro-chemo-mechanical response [107]. These two phenomena take place simultaneously and their interplay determines system-releasing efficiency. The polymer nanostructure also impacts the rate-limiting mechanisms of the ion diffusion and exchange process [65]. Cui investigated the impact of the morphology of substrate electrodes on electrically controlled drug release from PPy [150]. Fluorescein acted as a model drug, playing the role of the doping ion, while gold electrodes were covered with platinum to elevate the surface roughness of the substrate. All measured parameters, like the range of electrical stimulation of fluorescein release from the electrode, release per charge accumulation used during electropolymerization, and release per charge injected during electrical stimulation, confirmed an increase in the release of the drug from the material of higher roughness [150]. The drug loading capacity in electrically stimulated DDS is relatively low and there are different concepts concerning how to increase it. The group of Ge [111] proposed a PPy nanowire network on gold with micro- and nanogaps utilized as reservoirs to store drugs. In the system, drug loading capacity was dependent on the volume of the vacancies instead of the classic doping level. The author tested both hydrophilic (adenosine triphosphate, ATP) and lipophilic (dexamethasone, Dex) drugs and proved that both types are prone to be loaded due to the amphiphilic nature of the PPy matrix [111]. A SEM micrograph of PPy/oxacillin formed potentiostatically (at 0.80 V vs. SCE) presented a smooth surface [151] similar to the layer deposited in the presence of ionic liquids [35]. The lack of the typical cauliflower structure provides the conclusion that it is the concentration of oxacillin accompanied by its conversion to a protonated form that reduces the rate of electropolymerization [151].

Drug-delivery systems composed of a modified polypyrrole film with diminished ion exchange of the stored compound were proposed by Arbabian [152]. A millimeter-sized implant electronically controlled the drug release and was powered ultrasonically. Fluorescein-loaded polypyrrole nanoparticles were synthesized with a micelle-templated method and subsequently aerosol spray-coated onto the modified screen-printed electrode. The morphological analysis showed the high surface area of the resulting nanoparticulate film with pore sizes that enhanced drug loading and facilitated the release act [152]. 

**Figure 9:**
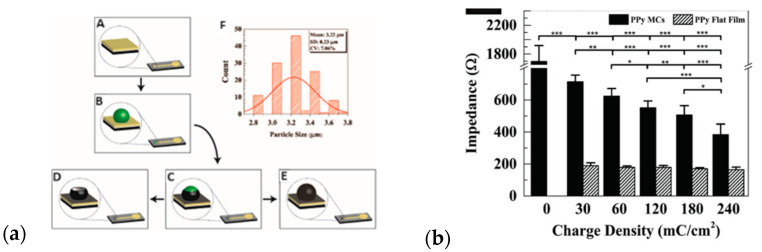
(**a**) Schematic illustration of the PPy MC fabrication process: (**A**) Au electrodes before surface modification, (**B**) electrosprayed PLGA microspheres on electrodes, (**C**) partial PPy encapsulation of PLGA microspheres, (**D**) PPy MCs formed by dissolving PLGA microspheres, (**E**) full PPy encapsulation of PLGA microspheres, (**F**) histogram of PLGA diameter distribution. (**b**) electrical properties of Au electrodes modified with PPy MCs: impedance at 110 Hz as a function of deposition charge density for PPy film/MCs (sold back) and PPy film without MCs (hatched gray), ***, **, and * demonstrate significant deference *p* < 0.001, *p* < 0.01, *p* < 0.05 between the groups. (reprinted from [153] with permission from John Wiley and Sons).

The polymerization of Py to nanostructural forms can be guided by a template method that is either soft or hard [64]. In electrochemical polymerization, the hard template is conductive or covered with a conductive surface. In the process, polymers with defined micro- or nanostructures are obtained. In the subsequent removal step, the template can be dissolved, leaving an imprint of the material. Abidian utilized such a procedure to fabricate monodispersed conducting polymer microcups [153]. The author presented the possibility of changing the physical surface properties of microcups to steer electrical properties and dexamethasone-loading/release characteristics (Figure 9a). SEM images revealed that only the highest value of deposition charge densities (240 mC/cm^2^) allowed for the full coating of the PLGA microspheres with PPy. Still, the dissolution of the PLGA template left partially shaped microspheres named microcups (MCs). The impedance of Au electrodes was markedly reduced after coating with PPy film/MCs (Figure 9b); however, still, the measured impedance of modified electrodes contained components of both PPy film and PPy MCs [153].

Biocompatible nanostructured conductive heparin-doped polypyrrole film was used as a high-capacity cation exchanger for the triggered release of chlorpromazine (CPZ) [154] with thermal and electrical dual stimulation. SEM images of the PPy–Hep–CPZ and PPy–Hep polymers prepared electrochemically possess a homogeneous, porous nanostructure with spherical morphology. The film structure markedly influenced the surface area due to the porous nanostructure, which increased the drug loading efficiency. The compact structure of the polymer was blamed for hampering the drug release. The immobilization and release of two model drugs, namely quercetin and ciprofloxacin, were proposed by Krukiewicz [155]. Two routes of immobilization were analyzed with drug incorporation at the stage of polymerization or post-synthetically. Both the immobilization method and the nature of the drug molecule influenced the surface morphology. The matrix involved in the reductive/oxidizing treatment was less uniform in comparison to the one obtained in in-situ synthesis. It was pointed out that the formation of large PPy grains and a rough surface was governed by the ion-exchange processes, namely the extraction of pristine doping anions from the surface followed by secondary anion incorporation along with the oxidation process.

#### 4.1.4. S as Sensors and Sorbents

**Figure 10:**
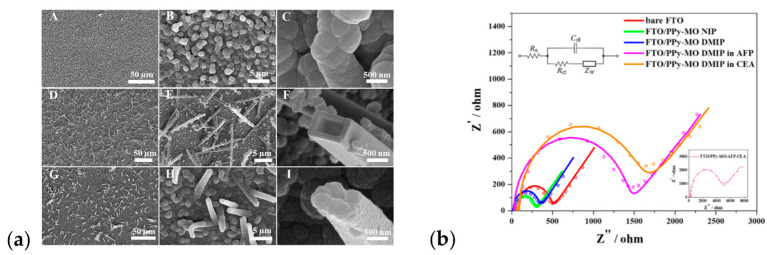
(**a**) FE-SEM images of electropolymerized coatings of (**A**–**C**) PPy, (**D**–**F**) PPy-MO NIP, and (**G**–**I**) PPy-MO DMIP FTO electrodes, (**b**) EIS spectra for deposited layers. (reprinted from [42] with permission from Elsevier).

The use of conjugated polymers for sensing applications is beneficial, as the intrinsic conductivity of matrices provides a tool for fabricating highly sensitive chemoresistive sensors. Numerous studies of sensors have been reported [156,157,158,159], with polypyrrole being one of the most promising in the field because of its stability and biocompatibility. Multiple available electrochemical deposition techniques are also an advantage. Cyclic voltammetry at different scan rates (5–50 mV/s) was utilized for the synthesis of polypyrrole on a gold electrode surface [160]. Deposited material was used for the electrochemical reduction in the simulant of nerve agents, namely dimethyl methyl phosphonate (DMMP), in an aqueous environment. SEM image analysis pointed to correlation of scan rate with surface morphology, showing globular, growing bud, hook, or rod structures. A decreased value of R_et_ (the electron transfer resistance) was reported for electrodes modified at 10 mV/s manifested in an extended surface area that enhanced electron transfer in the thin film of PPy [160]. The functionality of a dual-template molecularly imprinted polymer (DMIP) as a sensing layer to alpha-fetoprotein (AFP) and a carcinoembryonic antigen (CEA) as a lung cancer biomarker was verified by Bagheri [42]. PPy deposited on a fluorine-doped tin oxide (FTO) electrode served as an artificial antibody-like system. The characteristic morphology was a non-grown globular structure with an average diameter of particles of 1100 nm (Figure 10a). The presence of a template molecule (methylene orange, MO) induced organization, with a surface morphology of periodically dispersed hollow rectangular nanotubes. In the case of PPy-MO DMIP, the coating has a rougher structure dictated by the presence of biomarkers at the stage of electropolymerization. The addition of AFP (or CEA) influenced template formation due to the interaction with MO, e.g., by hydrogen bonding, and varied the polymeric structure of the imprinted layer [42]. Impedimetric detection served as a tool for rebinding the template detected with the charge transfer resistance, which increased as the concentration of AFP and CEA increased (Figure 10b).

An elegant immunosensor for the detection of anti-transglutaminase antibodies was constructed utilizing an overoxidized polypyrrole matrix [161]. A transglutaminase (tTG)-specific antibody represents a specific biological marker for coeliac disease. In the process, the transglutaminase antigen was deposited on a polymer, and gold nanoparticles covered a glassy carbon electrode. With the use of the EIS, the linear relationship between charge transfer resistance and analyte concentration was established. The SEM images of the AuNP and oPPy composite showed the transformation of material in the course of the overoxidation process—from a flake-like C_4_^−^-doped system to a typical “cauliflower-like” structure. The good surface coverage of the film surface with AuNP was noted [161]. The application of the sensor was verified using a calibration curve for known antibody concentrations.

A polypyrrole-covered platinum electrode was prepared to quantify dopamine [162]. The construct was built with layers of standard and overoxidized doped polypyrrole modified with AgNPs stabilized with chitosan. The surface morphology of the standard film showed its smooth surface, while a cauliflower morphology was found for the overoxidized one. The morphology of the PPy films displayed micro-spherical grains, with the size of the grains being in the range of 7–10 mm. Polypyrrole layers electrochemically deposited on the ITO-coated glass in the solution of chosen phenothiazine (PT) derivatives, namely methylene blue (MB), azure A (AA), and thionine (TH), were studied by Ramanavicius [158]. PT-based compounds are biologically active and electrochemically active. The coatings were electrochromic in nature, changing color at different potentials, and were sensitive to both pH value and ascorbic acid concentration. The surface morphology pointed out that the surface of the PPy-PAA layer was the most unevenly distributed, related to large polymer agglomerates of various sizes. For the PMB-doped layer, fairly smooth but pleated structures were formed, while the surface of the PTH-doped system consisted of larger grains, with an average particle size of 6 μm. The AFM data showed that the roughness of the surface decreased in the line for the PAA > PMB > PTH system, with visible wrinkles on the surfaces of the PTH and PMB dopants prescribed for the drying process [158].

**Figure 11:**
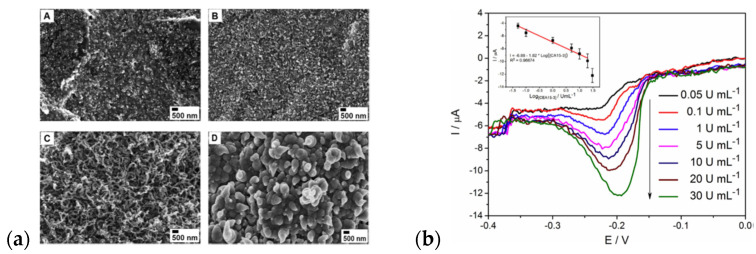
(**a**) SEM images of PPy deposited under polarization at E = 0.75 V/500 s from LiClO_4_ solution: (**A**) 1 mM; (**B**) 5 mM; (**C**) 10 mM; and (**D**) 15 mM. (**b**) Typical DPVs of immunosensors in PBS containing 0.1 mM H_2_O_2_ and 1 mM HQ with CA 15-3 antigen at different concentrations (from 0.05 to 30 U mL^−1^) and the calibration curve (inset) for CA 15-3 (reprinted from [163] with permission from Elsevier.

A disposable screen-printing carbon-ink electrode was covered by an electroactive bilayer film in the work reported by Nguyen [163]. The inner layer is composed of polypyrrole nanowires with a large surface-to-volume ratio and high conductivity in the neutral medium, while the outer layer is comprised of poly(1,5-diaminonaphthalene) (P(1,5DAN))-containing free amino groups. The accessibility of these groups is the perspective for coupling the further biomolecules. The modified sandwich-type electrode served as an electrochemical immunosensor for detecting breast cancer biomarkers (CA 15-3 antigen). The observed morphology changes strongly depended on the polymerization conditions, especially LiClO_4_ concentrations (Figure 11a). A concentration as high as 10 mM had to be prepared to obtain a steady increase in the anodic current response. However, a higher concentration (15 mM) provided a profound current increase, leading to a PPy cauliflower-type morphology. These observations are consistent with the description delivered by Fakhry [164] and Debiemme-Chouvy [104,165], pointing to the role of oxygen nanobubbles in layer formation. Differential pulse voltammetry was used as a recording tool for the quantification of the CA 15-3 antigen. The amplified current responses increased with analyte concentration and showed a linear relationship in the range of 0.05–20 U/mL (Figure 11b), proving the usability of the sensor [163]. 

A nanohybrid film of carboxylated polypyrrole and amine nanoclay was prepared as an immunosensor for the label-free detection of the human cardiac troponin T (cTnT) [166]. The nanohybrid film was formed in situ on the surface of the glassy carbon electrode, followed by the covalent immobilization of anti-troponin T antibodies using glutaraldehyde. The morphology of the film showed agglomerates of different two-dimensional laminar shapes. An interesting review focusing on the use of polypyrrole-based electrochemical biosensors for the diagnosis of colorectal cancer was provided by Wang [167] pointing to opportunities and challenges related to the use of PPy-based sensors for diagnosing colorectal cancer (CRC).

The electrosorption/electrodesorption process was utilized for the detection of salicylic acid (SA) with the aid of electrochemically controlled solid-phase extraction (EC-SPME) [168]. Nitrate-doped polypyrrole was a sorbent for the extraction of SA in plasma and urine samples. A rod-shaped stainless-steel electrode was covered with the vertically grown nanosheets deposited with the CV method. The morphology of the nanostructure PPy/nitrate sorbent revealed the presence of nano-sheets with a diameter of 14 nm, pointing toward a more porous, three-dimensional structure in comparison to the SDBS-doped system. It was validated to be more efficient for the electrosorption of chosen analytes [168]. In the work of Buszewski, [169] polypyrrole- and polythiophene-based SPME coatings were tested for the extraction of adrenolytic drugs (like metoprolol, oxprenolol, mexiletine, propranolol, propaphenon) from standard solutions and human plasma samples. The authors reported the impact of extraction time, desorption conditions, and pH on the sorption process of polysaccharide fibers. The SEM images of the fibers proved a more porous structure for PTh coating, which contributed to an increase in the extraction capacity relative to a more compact PPy one [169].

**Figure 12:**
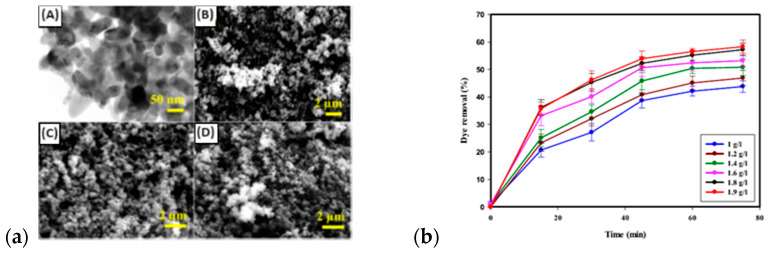
(**a**) Microscopic images of PPy/Ze composite sample: (**A**) TEM images of PPy/Ze composite, (**B**) SEM images of PPy/Ze composite, (**C**,**D**) SEM images of PPy/Ze composite after shaking with RB (**C**) or RR (**D**); (**b**) sorption curve for RB (Reactive blue) adsorption onto PPy/Ze nanocomposite. (reprinted from [171] with permission from Elsevier.

CPs are regarded as promising materials for monitoring environmental pollution, as CP-covered electrodes have been tested for the selective recognition of heavy metal ions in water [170]. The efficiency of an electrosensing electrode material relies on several parameters like the type of dopant; the modification of the polymer backbone, e.g., by chelating groups; or the preparation of an ion-imprinted matrix. The nanostructuration of sorbent material improved the sensitivity of sensors for quantitative and rapid analyses. A polypyrrole/zeolite nanocomposite was proposed as a nanoadsorbent for reactive dye, namely reactive blue (RB) and reactive red (RR), removal from synthetic solution [171]. PPy/Ze nanocomposite particles were agglomerated with a spherical shape (Figure 12) with an average size of 40–80 nm that was not changed after the adsorption of dyes. 

Also, a biocomposite composed of chitosan, starch, and polypyrrole with a sugarcane bagasse was utilized for the efficient removal of acid black dye [172]. Polypyrrole-modified copper electrodes were applied as a component of a lab-scale continuous flow system for the disinfection of well water contaminated with *Escherichia coli* [144]. The morphology of the modified PPy films showed microtube formation, which was not changed in the testing conditions, while the bacteria-killing process was monitored. Efficient transition-metal oxyhydroxide bifunctional electrocatalysts for water splitting made of phytic acid-doped polypyrrole nanotunnels with luminal-abluminal NiCo-(oxy)hydroxide nanosheets on a carbon cloth were proposed [173]. The presence of PPy ensured high mass loading and provided rapid electron/charge transportation during the electrolysis of water. Phosphate groups of organic acid acted as cross-linking sites for the metal ions, leading to a confining effect in terms of the migration and aggregation, resulting in the homogeneous dispersion of metal ions. The table below (Table 1) contains synthesis procedures and core characterization parameters for the discussed materials.

### 4.2. The Impact of Morphology on the Technological Applicability of PPy

#### 4.2.1. P as Corrosion Protection

Corrosion is a continuous obstacle that occurs when metallic substrates are in use. Different materials and approaches were proposed to decrease its impact as well as improve anticorrosion properties. For CP-coated substrates, several protection mechanisms were described, namely anodic passivation, cathodic protection, barrier protection, and a controlled inhibitor release model. Enhanced anticorrosive characteristics were shown for coatings electrodeposited from a solution of pyrrole and oxalic acid on an iron surface [175]. The acid additive provoked the growth of a protective layer composed of iron oxalate, preventing the anodic dissolution of substrate at the applied potentials. The protective properties of PPy-based coatings can be improved by the application of chosen electrodeposition parameters, including the type of doping anion and the construction of a multi-component or multilayer system [82]. For PPy films deposited from sodium salicylate solution synthesized on Ti-6Al-4V alloy potentiostatically [86], Zn was immobilized with two methods in the polymer to both prevent corrosion and deplete microbial growth. SEM micrographs showed the presence of hollow rectangular-sectioned microtubes for PPy deposited in salicylate solution, while the addition of Zn^2+^ ions distorted this shape to some extent, with deposits of zinc salicylate on the top. Both unmodified and modified films delivered corrosion protection by suppressing the active dissolution process in artificial saliva [86]. Similar anodic protection behavior was reported for PPy electrosynthesised on a nickel substrate [88]. Moreover, the adherence of the films markedly increased in this study. The electropolymerization of pyrrole in aqueous solutions of salicylate (0.5 M) was reported by Saidman [176]. The coating morphology presented hollow rectangular-sectioned microtubes. The bilayer system composed of differently doped PPy (underlayer doped with molybdate and nitrate and top layer doped with salicylate) was constructed to test anticorrosive properties. The inner and the outer layers were electrosynthesised at 0.8 V for 180 and 600 s, respectively. It was shown that bilayers were capable of protecting the substrate (316 L SS steel) against uniform and pitting corrosion during prolonged exposure [176]. Mild steel (MS) was covered with homopolymer and bilayer coatings composed of poly(N-methylaniline) (PNMA) and polypyrrole–dodecylsulfate (PPy-DS) using the potentiodynamic method [177]. Th surface morphology of homopolymer and bilayer coatings revealed by SEM images provided a cauliflower-like structure for PPy-DS synthesized in a narrow potential range (0.3–0.9 V vs. Ag/AgCl), while for the extended range (0.3–1.0 V vs. Ag/AgCl), the fiber-shaped fringes were visible. The PNMA/PPy-DS bilayer was found as the most corrosion-resistant at all immersion times (tested up to 240 h) [177]. The highly anticorrosive behavior and long-term stability of the dodecylsulfate-doped PPy system were confirmed by Syugaev [82]. Coatings composed of polypyrrole were deposited on zinc-coated steel by ultrasound (US)-aided electrosynthesis [46] and were tested as a physical barrier against corrosive species. US application provided more compact and homogeneous surface structures. Additionally, a more homogeneous distribution of doping ions (molybdate anions) within the film was noted with improved corrosion protection detected for sonicated films in comparison to commercial passivation systems containing Cr(III) and Cr(VI) ions [46]. Understanding the path for the deposition process on a zinc substrate enabled the construction of multifunctional high-powered sources for smart contact lenses [178]. The PPy was deposited at the receiver–lens interface on the back side of the Zn antenna to construct a hybrid power source for wearable electronics applications.

The corrosion behavior was related to the size and alignment of dopants in the polymer skeleton [31]. The presence of an additional benzene ring in the p-TSA and SDBS provided extra protection from chloride due to the formation of a barrier based on lamellas. The coating morphology showed a typical granular structure with modified compactness and uniformity enhanced for long-chain doped polymers (SDS and SDBS). This was prescribed to the presence of an extended carbon chain in these doping anions, which could also perform as corrosion inhibitors [31].

The system delegated for anticorrosion protection is realized also in the form of smart self-healing coatings [2]. An intrinsically conducting polymer formed an interlayer between the metal substrate and the top coating, which enhanced the spreading of the signal and transported active agents toward the defect. Such nano-containers with ICP were used as intelligent self-healing coatings for PPy doped with β-cyclodextrine (β-CD), benzotriazole (BTA), or 8-hydroxyquinoline (8-HQ) deposited in the presence of 3-nitrosalicylate [2]. A large passivation effect was reported, reflecting a synergy between the inhibitors and the re-oxidation of the PPy, accompanied by delamination decline. The morphology of the PPy deposited galvanostatically on the Zn substrate consisted of a globular structure with small spherical grains. The incorporation of additives brought about either no changes in morphology (for β-CD) or deviations in the inhomogeneity of the layer or the extent of agglomeration (for BTA and 8-HQ) [2]. Protective PPy films were synthesized under constant current control on the copper substrate from an aqueous phytate solution. The dopant-to-monomer ratio was equal to 1:5, while the solution pH was sustained at 6.0 to provide effective protection for the substrate. The corrosion test in 3.5% NaCl solution showed that the phytate-doped layer provided the passive state of the substrate to maintain for a time longer than 750 h [72]. The surface morphology of the IP6-doped PPy showed a typical globular morphology with particle sizes up to 2 μm in diameter. A higher current density applied in synthesis induced the conglomeration of material with clusters 5–10 μm in size. Polypyrrole deposited electrochemically on 304 stainless-steel (SS) surfaces was studied in the work of Jaouhari [179], where sodium phthalate and sodium saccharinate were compared as a dopant sources. The coatings showed good protection against the corrosion of the substrate in saline medium (3% NaCl), with PPy as a barrier against the penetration of chloride ions and as an oxygen reduction catalyst. The morphology of the coatings deposited with the galvanostatic method in a phthalate medium showed a homogeneous and compact distribution with a globular structure [179]. The effect of solvents with different donor abilities on the corrosion protection ability of PPy synthesized in potentiostatic mode on a stainless-steel support was traced by Li [180]. The films showed a spherical structure, observed in SEM images, and good corrosion protection ability was also found for materials prepared in nitromethane solutions, which was most promising. It was pointed out that this solvent provided only a weak interaction with the intermediate species formed during polymerization. As a result, the most compact accumulation and the highest conductivity were found [180]. The application of synthesized anticorrosion coatings and choosing a rational corrosion inhibitor to the PPy coating, coupled with the intelligent inhibitor release system, help to bring the PPy-based system into common use. Still, their mechanical properties shall be improved.

#### 4.2.2. M as Mechanical Aspects

Π-conjugated polymers combine electronic functionality with mechanical robustness. The materials were tested for their stretchability and flexibility [181], which are required for application in fields like wearable health monitors [182], stretchable electrochemical sensors for cell and tissue detection [183], neurological recording [184], or soft electronics [185]. In [186], the authors analyzed the stress-strain data from pull tests on conjugated polymers to explore the molecular and microstructural parameters that influence mechanical properties. Studies of mechanical properties, namely reduced elastic modulus, indentation hardness, and creep, were reported by Başman [187] for PPy on a Pt working electrode. Data derived from the depth-sensing indentation (DSI) technique showed that a rise in the concentration of support electrolytes reduced both elastic modulus and indentation hardness values. This was correlated with an increase in the free volume accompanying higher doping levels. Additionally, it also resulted in the enhanced creep of the samples [187]. Spinks [84] obtained a material of high conductivity and superior mechanical properties with the modification of the synthetic path, where several washing steps were added. The alternative procedure allowed for a decrease in the concentration of oligomeric species in the close vicinity of the electrode, thus hindering the three-dimensional growth, leading to a more compact layer. For thin films of PPy (100 nm) doped with PSS, an increased strain and strain rate were observed [64]. These mechanical properties are important in the field of mechanical actuation and are correlated with volume changes, strain, strain rates, and final work efficiency. The rate of actuation for CP is primarily limited by the volumetric charging rate and mass diffusion, which evaluates the diffusion time for ion movement within the polymer [64,107]. The composite based on cellulose nanopaper (CNP) with chitosan and PPy granules in the presence of polysaccharide improved the mechanical properties in a dry state. However, in the CNP/PPy blend, a decrease in mechanical strength was reported as a result of the tendency to break hydrogen bonds within CNFs due to the presence of PPy [148]. For the BC (bacterial cellulose)/PPy composite, it was reported that tensile properties were enhanced by the step “dry-rewet” method. The brittleness of the dry composite was reduced after the rewetting step [133]. Also, a higher elongation at break was reported, which responded to weakened Van der Waals forces between BC/PPy fibers due to water molecules, leading to the increased mobility of the fibers in the dry-rewet material.

#### 4.2.3. B as Bubbles and Nanoporous Structures

It is possible to form empty nanostructured containers; this is realized by the gas-assisted technique. In the first stage, gas bubbles are inhaled into micelles and adsorbed on the surface of the substrate. In the subsequent step, these structures play the role of a template for PPy deposition [188], which takes the shape of spheres, bowls, and cups. The technique is free from the template removal stage, in opposition to aromatic surfactants like β-naphthalene sulfonic acid (NSA) [189] and poly(styrene sulfonic acid) (PSSA) [190] that are most frequently used. The arrangement forces are mainly dictated by synthetic conditions like vertex potential, the surfactant’s concentration, the number of cyclic scans, and solution pH.

**Figure 13:**
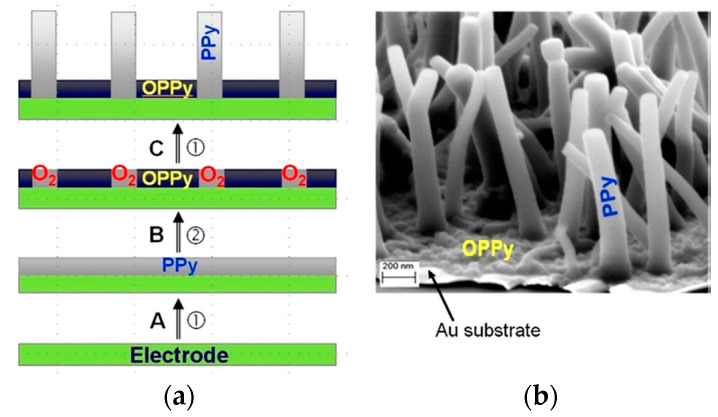
(**a**) Schematic model showing the process of PPy nanowire electrogeneration under potentiostatic conditions. A: deposition of an ultra-thin film; B: generation of OH^−^ and of O_2_ nanobubbles; C: growth of the PPy nanowires. Reactions (1): Py oxidation; (2): water oxidation; (**b**) SEM micrograph of PPy deposited at 0.75 V/SCE in 0.15 M Py + 0.2 M K_2_HPO_4_ + 10^−3^ M LiClO_4_ on Au/mica substrate. (reprinted from [164] with permission from Elsevier).

Turco provided interesting templates for the synthetic path of PPy nanowires [191] using non-static solution surface electropolymerization. A pivotal role in the deposition on an indium tin oxide-coated PET working electrode was prescribed for the oxidation of pyrrole along with oxygen nanobubble formation. The morphology of the layer was governed by synthetic parameters like flow rate, pH of the electrolyte solution, and time of process. Nanowires with a diameter in the range of 40–300 nm were obtained, with a larger electroactive area of the sample prepared at acidic (pH 6.8) conditions [191]. Picturesque structures were obtained by the use of a gas bubbles-based procedure in the work of Guittard [192], based on the application of thienothiophene derivatives. A set of nanotubes and tree-like structures were evaluated as highly adhesive to gold-coated silicon wafers that served as working electrodes. The author stressed that the water content is urgent for the proper formation of gas bubbles in the process, even in anhydrous dichloromethane. The mechanism of formation of electrogenerated PPy nanostructures based on the templateless method was evaluated by Debiemme-Chouvy [164]. The successful growth of the structurally ordered layer is dictated by the composition of electrolyte solution with the proper content of the phosphate-based anions, namely weak-acid monohydrogenophosphate and non-acidic perchlorate. Only with a high content of the second (c > 0.5 mM) were a superhydrophilic nanostructured conductive film of nanofibers or oriented nanowires (50–120 nm in diameter) deposited (Figure 13) [164,165].

Spontaneous nanostructuration was enabled by the oxidation of water followed by the production in hydroxyl radicals and nanobubbles of oxygen. Moreover, the authors proved that the nanobubbles shield the PPy film against the action of the hydroxyl radicals, which react with the polypyrrole film, leading to overoxidation [73,104].

Well-shaped nanostructures were provided in the study of McCarthy [193], where electrodeposition was performed on a glassy carbon rod substrate from an emulsion of pyrrole and its N-functionalized derivative, namely N-(2-cyanoethyl)-pyrrole. The emulsion was prepared by two sonication procedures that resulted in various microstructure formations. The polymer was shaped as aligned open- and closed-pore microtubes, where adsorbed toluene droplets played the role of soft templates supporting polymer growth. The rate of polymer propagation was controlled by the chemical composition of the electrolyte solution, with the ClO_4_^−^ and H_2_PO_4_^−^ doping ion concentration being the most appropriate factor [193]. Polypyrrole-coated pickering-type droplets were studied as light-responsive carriers of oily material [194], leading to the light-driven remote motion control of the droplets. A template-based method for preparing nanoporous PPy on highly oriented pyrolytic graphite (HOPG) was reported by Hu [195]. The size and distribution of the hydrogen nanobubbles were observed and controlled by in-situ electrochemical atomic force microscopy (EC-AFM). The structure of the film was adjusted by the choice of controlled parameters, like the applied potentials and time. The depth of the nanopores in the PPy film depended on the number of cyclic voltammetric scans for polymer deposition, which were 4 nm and 8 nm for synthesized samples.

A porous diblock copolymer template, namely a copolymer of styrene and methyl methacrylate (PS-b-PMMA), was utilized in a procedure aimed at the deposition of high-density polypyrrole nanorods on an indium-tin-oxide (ITO)-coated glass electrode [196]. The nanorods were characterized with distinctly higher conductivity in comparison to thin PPy films, which reflected their high degree of chain orientation coming from the synthetic stage, where the growing chains were confined into nanosized cylindrical cavities. When the pore sizes of the templates were more than 50 nm, the nanotubes were formed, while for smaller diameters (<25 nm), nanorods were constructed [196]. The confinement effect in the environmentally friendly synthesis of PPy within advanced polymeric templates was traced by Malardier-Jugroot [197]. Two polymeric templates with different functional groups were used, namely SMA (poly(styrene-alt-maleic anhydride)) and IMA (isobutylene-alt-maleic anhydride)). In the water environment, SMA produced amphiphilic nanotubes with hydrophilic shells and hydrophobic cavities, while IMA produced amphiphilic lamellae sheets with a hydrophilic outer layer and a hydrophobic interior. The chemistry of the templates did not markedly influence the reaction, while the confinement effect was proven to be crucial for the reaction (in block copolymer PS-b-PAA templates, with diameters of 40 to 70 nm, the polymerization did not occur) [197]. Polycarbonate particle track-etched membranes with different pore sizes were used for PPy electrodeposition by Demoustier-Champagnen [98,198]. A metallic bilayer composed of Cr and Au was evaporated on the membrane and served as a working electrode. As a result, nanotubules were observed, with their thickness dependent on the pore diameter of the template membrane as well as the nature of the doping agents: perchlorate (ClO_4_^−^), dodecyl sulfate (DS^−^), toluenesulfonate (TS^−^) or polystyrenesulfonate (PSS). Additionally, the relative conjugation length in the PPy chains increased with decreasing pore size [198], and kinetics studies were performed [98]. A whelk-like helix composed of PPy deposited on a glassy carbon (GC) electrode was presented by Jiujun Zhan [77]. The material was highly doped with sodium dodecyl sulfate (doping level of 0.612), with the SDS concentration reflecting a profound effect on the morphology. The surface enhancement of the electropolymerized PPy whelk layer was determined by the use of an electrochemically active probe, namely K_3_Fe(CN)_6_. The average peak current on the CV for the whelk modified layer was roughly six times higher in comparison to a bare GC electrode and four times higher in comparison to a normal PPy-covered GC electrode, proving the enhancing power of the modified film to the redox reactions [77]. The morphology of polypyrrole nanostructures was the subject of interest of Ramanavicius [199]. A pre-adsorbed layer of pyrrole on the surface gold electrode before polymerization allowed for single pulse-potential electropolymerization with nanostructured layer formation. Aggregated spherical particles with 50 nm diameter and unequally distributed conductivity were deposited. The material is composed of a mixture of more conducting areas and a small-sized PPy of lower conductivity.

#### 4.2.4. C as Carbon-Based Materials

In many cases, the good properties of polypyrrole can be even better when a proper other ingredient is introduced to prepare a multi-component system. This seems to be true for the “marriage” of polypyrrole and carbon-based materials, where attempts to provide new materials are highly successful and promising. Different carbon-based substrates (vitreous carbon and Au (111)) as well as various experimental conditions (dynamic vs. static potential protocols) and halogen dopants (I^−^ and F^−^) were tested in the work of Batina [200]. The morphological study revealed that the conditions induced ring (doughnuts) and microcontainer formation. The microstructure formation was contingent on the occurrence of the overoxidation of PPy [200]. The polypyrrole morphology showed significant influence on the BET surface in the studies of Mosch [201], where the porosity and surface measurements revealed the increase in average pore size for composites in comparison to pure carbons (either CB or MWCNT). The highest electrochemical area and mesoporous structure belonged to composite samples, which were also characterized by increased capacitance. It revealed the beneficial aspect of the deposition of polymers on the carbon substrate [201]. The electrochemical capacitance of composite coatings composed of PPy and carbon nanotubes (CNTs) was also investigated in supercapacitor application by Bara [202]. PPy deposited on CNTs on a Ni catalyst layer deposited on a Si/SiO_2_ substrate showed uniform coverage with high capacitance in an acid electrolyte [202]. A morphological analysis of PPy/(f-)MWCNT composites was provided by Zak [203], where materials were prepared with either pristine or functionalized multi-walled carbon nanotubes (MWCNTs), either oxidized (MWCNT-Ox) or pyrrole-modified (MWCNT-Py). The surface morphology of PPy/MWCNT revealed polypyrrole-covered tubular nanoparticles with regular structures determined as crystal domains, originating from the orientation of growing PPy chains. The surface functionalized scaffolds lost stiffness and did not induce crystallization that efficiently; hence, images of PPy/MWCNT-Py and PPy/MWCNT-Ox presented less oriented structures. The morphology of the polymer deposited electrochemically in the presence of functionalized MWCNT is dependent on the extent of coverage of the nanotubes by functional groups and hydrophilicity [203]. Protective films composed of polypyrrole doped with dodecylbenzene sulfonate (DBS) for a copper substrate were proposed by Breslin [204]. The film was enriched with carbon nanotubes (CNTs) and the system was deposited on a pre-layer composed of tartrate-doped PPy. In terms of morphology, the cauliflower structures were smaller when CNTs were present, provoking a more disorganized structure of the polymer. The CNTs served as an additional scaffold for the further growth of the polymer, with the chains interconnecting with the CNTs. The concentration of Cu^2+^ ions manifesting the dissolution of corroding copper was observed for the polymer-modified system. The protection activity was prescribed to the formation of large anionic micelles as well as the presence of CNTs imparting a negative charge to the surface, which became repellent to chloride anions. A poly(vinylidene fluoride) (PVDF)/multi-wall carbon nanotube (MWCNT)/polypyrrole (PPy) composite was prepared and tested as an ultrafiltration membrane for the removal of crude oil from refinery wastewater [205]. Morphological characterization showed that PPy grew at the MWCNTs with different thicknesses—lower amounts of MWCNTs allowed for thicker PPy layers located at the surface of carbon-based material, which led to enhanced surface roughness. Cross-sectional images revealed larger pores for PPy-modified membranes responsible for the improved water permeability [205]. Biomimetic sensors for the residues of an anticancer drug, namely methotrexate, were constructed [206]. Molecularly imprinted polymer (MIP) polypyrrole electrodeposited with cyclic voltammetry on a glassy carbon electrode (GCE) incorporated multi-walled carbon nanotubes (MWCNTs). The synthetic procedure provoked the vertical growth of the polymer nanotubules that enlarged the number of electroactive sites, leading to enhanced sensitivity [206]. A set for the simultaneous microextraction and determination of heavy metals was proposed by Rohanifar [207]. A solid-phase microextraction (SPME) sorbent material was made of coating composites containing polypyrrole (on pencil lead) electropolymerized with carbon nanotubes (CNTs) and various metal-chelating ligands like 1,10 phenanthroline [207]. This was effective in the determination of silver, cadmium, cobalt, iron, nickel, lead, and zinc, mainly because of the high porosity and large surface volume that allows for the high extraction capacity of the analytes [207]. Polypyrrole/carbon nanotubes (PPy/CNTs) deposited electrochemically on stainless-steel meshes served as the solid-phase extraction sorbent [208]. It was used to extract environmental pollutants from water samples, like polycyclic aromatic hydrocarbons (PAHs). The CNT-containing sorbent efficiency was superior to pure PPy-coated stainless-steel meshes and C18 commercial cartridges. A desalination battery based on the PPyCl/CNT composite was proposed by Kong [94] with reversible ion storage in the material. A battery consisted of a PPy-Cl anode and an Na_0.44_MnO_2_ (NMO) cathode, with 1.0 M NaCl as the aqueous electrolyte. Cl-doped PPy completely covered the surface of the CNTs, leading to the increase in diameter of the CNTs with rod-like morphology.

#### 4.2.5. E as Energy Conversion Systems (Solar, Photothermal and Energy Storage Applications)

Conventional energy sources are limited in nature, so other solutions are sought and studied, including marine, nuclear, solar, bio, and wind resources. Solar energy is transmitted with solar thermal technology, photovoltaic energy conversion, and solar hydrogen gas production technology [209]. There are many technologies to convert solar energy; photovoltaics (PVs) is one of the cleanest to choose. Conjugated polymers are used for the fabrication of photovoltaic devices, namely electrochemical and dye-sensitized solar cell (DSSC) devices [210]. The overall efficiency of bulk heterojunction (BHJ) architecture photovoltaic cells depends to a large extent on the nanomorphology of the photoactive layer. It can be tuned by processing parameters like the choice of solvent(s) in the spin-casting method, thermal and solvent annealing, solvent additive, and blend composition [210]. Molecularly imprinted polypyrrole was proposed as counter-electrode material for dye-sensitized solar cells (DSSCs) by Sangiorgi [211]. Moreover, 2-aminoacetic acid (glycine) and L-2-aminopropionic acid (L-alanine) were used as template molecules at the imprinting stage. Gel-state DSSCs based on MIP-PPy CE with glycine were characterized by a 20% increase in the power conversion efficiency along with a 50% reduction in the charge transfer resistance, in comparison to the cells based on NIP-PPy. In terms of the morphology of MIP and NIP PPy electropolymerized on the FTO surface for glycine coating, they showed globular-shaped particles with circular nano-aggregates smaller than the ones found for NIP-PPy. At the same time, for MIP with L-alanine, the presence of small aggregates with needle-like shapes was visible, forming more homogenous film. The differences were prescribed to various molecular surface areas of the two template molecules [211]. Carbon fabric (CF) coated with polypyrrole was used as a flexible counter electrode in DSSC [212]. It showed a homogenous structure along with proper electron/hole charge transfer. The power conversion efficiency (PCE) of the constructed DSSC reached 3.86%. The application of polyoxometalate (POMs: H_3_PW_12_O_40_) led to (PW12)-doped polypyrrole (PW12-PPy) hybrid film tested as an efficient counter electrode in a DSSC [213]. POMS exhibit multi-electronic reversible reactions and good electrochemical catalytic activity, resulting from transition metal oxide components. The average power conversion efficiency of the DSSC illuminated with solar radiation (PCE, 6.19%) was comparable with ordinary Pt-cathode devices. SEM images of the PW12-PPy showed a sphere-like structure with a large specific surface area [213].

**Figure 14:**
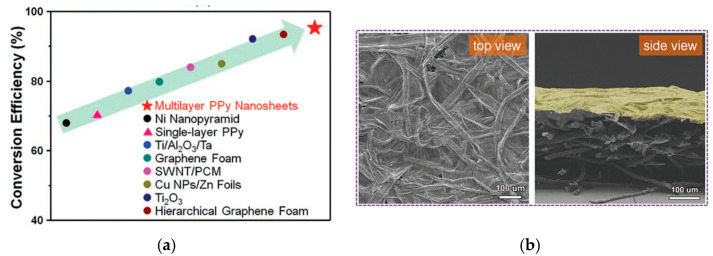
(**a**) Solar–thermal conversion performance of multilayer PPy nanosheets compared to recently reported high-performance solar–thermal materials; (**b**) multilayer PPy nanosheets—SEM images of the paper coated with one layer of PPy nanosheet. (reprinted from [214] with permission from John Wiley and Sons).

Converting solar energy into heat is an appealing idea to use based on the photothermal property and low thermal conductivity of PPy. A multilayer PPy nanosheet on paper substrates enhanced broadband and wide-angle light absorption across the full solar spectrum, resulting in a solar–thermal conversion efficiency of 95.33% (Figure 14a) [214]. On the sample surface, structures like wrinkles and ridges were formed spontaneously (Figure 14b), while their number became greater as the number of PPy layers increased. The average height of surface features increased gradually from 5 μm to more than 50 μm as the number of PPy nanosheets increased [214]. 

Chemical vapor deposition polymerization (CVDP) was utilized to achieve a dark PPy layer on different substrates that functionalized as a light absorber to convert light to heat. It was subsequently used for solar-driven interfacial water evaporation procedures [215,216,217]. It delivers a solar-driven evaporation strategy for clean water production. The membranes can obtain a high stagnation temperature (up to 82.3 °C under one sun illumination (1 kW/m^2^)) [215].

Photothermal therapy (PTT) uses localized heat derived from light-absorbing materials being subjected to near-infrared (NIR) laser radiation. It is a new tool for the thermal ablation of cancer cells with a noninvasive therapeutic modality. Two-dimensional ultrathin polypyrrole nanosheets synthesized using a space-confined protocol were tested as broadband absorption materials for prospective use as photothermal agents (PTAs) in the second NIR window (1000−1350 nm) [20]. The layered lamellar structure of the material was shown by SEM images. The photothermal conversion efficiency achieved 64.6%, surpassing previous PTAs that are active in the second NIR window. Good biocompatibility and notable tumor ablation ability in the second NIR window were confirmed. A polypyrrole-based platform complexing with DL-menthol (DLM) was also prescribed as a near-infrared light and thermo-responsive system for drug delivery [6]. The material generated bubbles under NIR light illumination, assuring control over encapsulated drug (diclofenac) release. Thermo-responsive nanogels enriched with the efficient near-infrared (NIR) transducing polypyrrole were tested for combinational photothermal and chemotherapeutic therapy along with photoacoustic imaging [218,219]. The electrochemical performance of polypyrrole makes it a useful material for rechargeable battery electrodes [220]. The calculated values of the specific charge of this polymer make it a promising positive electrode material while used in combination with a negative metallic electrode.

CP-based systems can be elegantly utilized as components of wearable electronics used to regulate human activities [221]. Among other things, one can point to piezoelectric nanogenerators (PNGs), which provide the opportunity to construct battery-free self-powered devices. Such constructs were proposed on PPy [222], PEDOT [221], and PANI [223], usually in the form of matts formed with an electrospun poly(vinylidene fluoride) (PVDF) nanofiber (NF) [221,223] and covered with a vapor-phase polymerized (VPP) material. After the process, PEDOT networks were observed both coating the surface of NFs and being inserted into the interspace of NFs [221]. The device performance assessed according to output voltage was 48 V for PEDOT- [221], 20 V for PPy- [222], and 10 V for PANI [223]-based PNG.

## 5. Conclusions

Polypyrrole is a significantly useful material derived from an inconspicuous pyrrole ring. The available synthetic procedures allow for the precise sculpturing of both the chemical composition and morphology of the forming polymer. Multiple variations shall be taken into consideration to take advantage of the synergy effect coming from the sophisticated nanostructuring of the material at the stage of choosing the polymer procedure (proper solvent, doping ion, substrate choice), during the polymerization (conditions like temperature, stirring, enhanced impulses, like ultrasounds) or at the post-synthetic functionalization stage. The scope of possibilities is greater for composite preparation where a multiphase system is produced. The scientist is supposed to be a designer who uses the right bricks, at the right time, with the right sequence, to obtain advanced materials with high utility. Usually, it is an application field that imposes the required properties and guides us to deliver, e.g., either a highly porous substrate for neural scaffolds or a homogeneous, compact coating for corrosion protection. As we have gained knowledge of the relation between the synthetic path and morphological properties, it is possible to use it in the reverse technology and tailor future materials. 

At present, the main challenges for the wide use of PPy-based materials are connected with inadequate long-term stability. Several parameters still need to be improved for practical applications, namely mechanical properties, adhesion to substrates, and long-term chemical/environmental stability, for most prospective applications in fields such as coated neural implants, batteries, and energy storage materials or highly sensitive and selective sensors. The future perspective of the usage of PPy-based materials is spreading, mainly in the field of scaffolds for regenerative medicine supported with hydrogel components; responsive drug-delivery modules; and charge storage systems for novel batteries. In each case, morphology is a crucial parameter that governs the material’s response in the working system. The process of morphology formation is an intermediate step between the basic reactions of macro-chain synthesis on the one hand and material performance on the other. Its understanding provides a bridge to embarking on a path toward improvements. Moreover, the future belongs to the composite structures, with the most effective structures being bioinspired, which provides even more possibilities to the sculpture of the nanostructure.

## Figures and Tables

**Table 1:** Synthesis procedures and characterization parameters for PPy-based materials applicable in the biomedical field.

Material	Synthesis Mode	Technique	Dopant/Initiator	Conductivity S/cm	Morphology	Application Filed	Source
block co-polymers of PPy with poly(ε-caprolactone) and poly(ethyl cyanoacrylate)	ChOP	two-step procedure: macromonomer formation and Py co-polymerization	*para*-toluene sulfonate (pTS^−^)	(18–32)	a flat compacted surface	cell proliferation platform (rat PC12 cells)	[120]
PLLA/PCL fibers coated with PPy and chitosan (CS)	EChP	WE: ITO with electrospun PLLA/PCL fibers galvanostatic (8 min/2 mA)	chloride (Cl^−^)	(1–1.1)·10^−2^		cell differentiation platform and neurite growth (PC12 cells)	[122]
hydrogel based on sodium alginate, gelatin and polypyrrole	ChOP	rapid mixing/−20 °C	ammonium persulfate (I)	(1.2–1.6)·10^−2^	network structure with well-dispersed polypyrrole particles	self-healing conductive hydrogels	[123]
aligned PPy/PLA composite electrospun films	ChOP	P123 used as a template dropwise method (18 °C/6 h)	aqueous FeCl_3_	4.6	spherical PPy particles	platform for differentiation of human cord mesenchymal stem cells	[124]
collagen-heparin-polypyrrole composite	ChOP	vigorous stirring for 30 min followed by standing at r.t	FeCl_3_	0.11–0.336	compact structure with partial orientation	neural scaffold in the application of peripheral nerve regeneration (PC12 cells cultured)	[174]
**Material**	**Synthesis Mode**	**Technique**	**Dopant/Initiator**	**Tested Strain**	**Morphology**	**Application Filed**	**Source**
branched polypyrrole	EChP	WE: anode (metal wire) potentiostatic (9 V/10 min)	sodium dodecylbenzenesulfonate (DBSA), cetyl trimethylammonium bromid (CTAB)	*S. aureus*, *E. coli*, *K. pneumoniae*	fractal structure	antibacterial material	[8]
polydimethylsiloxane (PDMS) gradient doped with PPy	ChOP	dropwise technique under continuous mixing/ 2.5 h at 150 rpm	FeCl_3_	*E. coli*	increased surface roughness with typical granular forms	switchable superhydrophobic and self-cleaning material with drug releasing ability	[137]
a duplex coating based on PPy and molybdate—originated layer loaded with silver	EChP	WE: AZ91D (magnesium alloy) potentiostatic (1.15 V/600 s for 0.50 M NaSa and 0.80 V/1800 s for 0.10 M NaSa)	sodium salicylate (NaSa)	*E. coli*	globular morphology for lower NaSa concentrations, rectangular microtubes for higher	antibacterial activity with anticorrosive performance	[138]
PPy with oxygen plasma immersion ion implantation (O-PIIi)	EChP	WE: Ti/galvanostatic (5 mA/cm^2^, 10 min), r.t.	TsONa (sodium p-toluenesulfonate)	*E. coli*, *S. aureus*	Cauliflower morphology, after O-PIII treatment—pit-like structure occurs	antibacterial material	[134]
nanostructured PPy	template-free EChP	WE: Ti/galvanostatic (0.9 mA/cm^2^, 5 min), r.t.	sulfosalicylic acid in PBS	*S. aureus*	oriented nanorods with large specific surface area	antibacterial material	[142]
**Material**	**Synthesis Mode**	**Technique**	**Dopant**	**Release Mode**	**Morphology**	**Application Filed**	**Source**
polypyrrole	EChP	WE: Pt-black coated glass/potentiostatic (0.7 V, 200 s)	fluorescein	10 s pulses/−2.0 V into PBS	typical globular morphology	drug-delivery module	[150]
polypyrrole nanowire	EChP	WE: Au electrode/ galvanostatic (0.477 mA/cm^2^, 1600 s) pTS^−^ in PBS (pH 7.40)	adenosine triphosphate (ATP) dexamethasone (Dex)	CV stimulation (−0.9:0.6) V	nanowire network with porous interwoven structures	drug-delivery module	[111]
oxacillin-doped PPy (PPyOx) PPyOx modified with chitosan	EChP	WE: gold, platinum titanium/potentiostatic (−0.80 V vs. SCE, 500 s)	oxacillin	constant potential at 0.30 V or 0.60 V	smooth polymer films with roughness induced by the oxacillin presence	drug-delivery module	[151]
nanostructu olysaccharideride-doped polypyrrole	EChP	WE: Pt Two-step procedure: pre-electropolymerization in hep presence, potentiostatic (+0.9 V vs. Ag/AgCl/100 s) and electropolymerization in the CPZ presence potentiostatic (+0.7 V vs. Ag/AgCl/900 s)	heparin sodium salt (50,000 units) chlorpromazine hydrochloride	OCP and constant potential conditions (0.1:0.4) V	homogeneous, porous nanostructure with spherical morphology	drug-delivery module	[154]
doped polypyrrole	ECHP	WE: platinum foil in-situ drug immobilization mode—cyclic voltammetry (CV) ex-situ drug immobilization—CV for polymerization of Py followed by oxidative immobilization of drugs	quercetin and ciprofloxacin	constant a reduction potential (−0.5 V vs. Ag/AgCl) in PBS	matrix obtained by ex-situ mode less uniform with larger PPy grains and rougher surface	drug-delivery module	[155]
**Material**	**Synthesis Mode**	**Technique**	**Detected Analyte**	**Detection Mode**	**Morphology**	**Application Filed**	**Source**
Doped polypyrrole	EChP	WE: gold electrode/sodium perchlorate CV with different scan rate (5:50) mV/s	dimethyl methyl phosphate (DMMP)	EIS	globular and rod (for slow sr) structures and packed globular system for high sr	sensor	[160]
Molecularly imprinted polypyrrole	template assisted EChP	WE: fluorine-tin oxide FTO/CV (10 cycles, (0.0:0.7) V, 50 mV/s) in PBS, pH = 7.2	carcinoembryonic antigen (CEA) alpha-fetoprotein (AFP)	EiS	PPy-MO NIP: hollow rectangular nanotubes PPy-MO DMIP: rougher tubular structure	sensor	[42]
Gold—overoxidized polypyrrole nanocomposite	ECHP	WE: glassy carbon electrode (GCE), LiClO_4_, potentiostatic (800 mV (vs. Ag/AgCl)/120 s, overoxidized at 1.0 V/420 s), AuNP- CV (0.2:−1.0) V, 50 mV/s, 15 cycles	tissue transglutaminase (tTG)-specific antigen	EIS	OPPy: cauliflower-like structure with good surface coverage of the AuNP	sensor	[161]
Poly(1,5-diaminonaphthalene)/polypyrrole bilayer	EChP guided with oxygen nanobubbles	WE: screen-printed electrodes (SPEs) Two-step procedure: electropolymerization of Py potentiostatically (0.75 V/500 s in 0.2 M Na_2_HPO_4_, LiClO_4_ (1:−15) mM) followed by P(1,5DAN) deposition, CV (50 mV/s, (−0.02:0.75 V)	CA 15-3 antigen	DPV	nanowires (for optimized dopant concentration), for high concentration cauliflower-like structure	sensor	[163]
Polypyrrole/Nanoclay Hybrid Film	EChP	WE: glassy carbon electrode (GCE) CV ((−0.10:1.0) V, 200 mV/s, 20 cycles)I ACN, 0.1 M LiClO_4_ Anti-cTnT antibodies immobilized with glutaraldehyde (GA)	cardiac troponins (T and I)	SWV	heterogenous film formed by agglomerates of two-dimensional laminar shapes	sensor	[166]

ChOP—chemical oxidative polymerization, EChP—electrochemical polymerization, PBS—phosphate buffer saline, CV—cyclic voltammetry, EIS—electrochemical impedance spectroscopy, sr—scan rate, DPV—differential pulse voltammetry, SWV—square wave voltammetry.

## Data Availability

No new data were created or analyzed in this study. Data sharing is not applicable to this article.
